# Urachal Mucinous Cystic Tumour of Low Malignant Potential With Incidental Clinical Presentation: A Case Report With Clinicopathologic Features and Review of the Literature

**DOI:** 10.1155/crip/5573633

**Published:** 2026-05-14

**Authors:** Alex Miller, Maneesha Saxena

**Affiliations:** ^1^ Department of Renal Medicine, Toowoomba Hospital, Darling Downs Hospital and Health Service, Toowoomba, Queensland, Australia; ^2^ Department of Anatomical Pathology, Royal Brisbane and Women’s Hospital, Pathology Queensland, Brisbane, Queensland, Australia, health.qld.gov.au

## Abstract

Urachal mucinous cystic tumour of low malignant potential (MCTLMP) is a rare cystic epithelial neoplasm of urachal origin, with fewer than 50 cases described as such in the English‐language scientific literature. This case report describes an instance of urachal MCTLMP discovered incidentally in a 59‐year‐old female patient following the performance of imaging studies for an unrelated condition: Management consisted of surgical resection of the tumour and partial cystectomy, with no complications and no recurrence after 10 months of follow‐up. Sections of the partial cystectomy specimen demonstrated histological features that were reminiscent of a low‐grade appendiceal neoplasm or a borderline ovarian mucinous neoplasm, which were favoured to represent urachal MCTLMP. This case report highlights the clinical importance of both the complete surgical resection of tumour and the exclusion of secondary involvement of the bladder or urachus by a glandular tumour for the diagnosis of urachal MCTLMP and represents the emerging ‘typical’ clinical trajectory of a patient with urachal MCTLMP. Ongoing longitudinal study is required to better understand the longer term behaviour and clinical course of urachal MCTLMP.

## 1. Introduction

Epithelial neoplasms of urachal origin are classified as per the World Health Organization Classification of Tumours [1]. Namely, urachal neoplasms can be divided into noncystic (or usual) tumours and cystic tumours: Urachal cystic tumours are subsequently categorised according to malignant potential and include mucinous cystadenoma, mucinous cystic tumour of low malignant potential (MCTLMP, with intraepithelial carcinoma or without intraepithelial carcinoma) and mucinous cystadenocarcinoma (microscopically invasive or frankly invasive) [1]. Histologically, urachal MCTLMP is characterised by a stratified cyst lined by no more than three layers of epithelial cells, with or without structural abnormalities including pseudopapillary or villous patterns [2]. Mild to moderate nuclear atypia may be seen, but mitoses are rare or absent, and there is no stromal invasion [2]. Elaboration of mucin into the cyst is common, as is calcification of the cyst: Ossification of the cyst is rare but has been described [3]. Morphologically, the lesion has been described as analogous to an atypical proliferative mucinous tumour of the ovary [4]. Urachal MCTLMP is often diagnosed based on these histological features alone, without the performance of immunohistochemical studies, but the majority of urachal mucinous cystic tumours have an intestinal phenotype, staining positively for CK20 and CDX2 and staining negatively for nuclear beta‐catenin, oestrogen receptor and progesterone receptor [5]. The genetic basis for the development of urachal neoplasms is not well understood presently: However, mutations of KRAS have been identified in cases of urachal mucinous neoplasm, and this may represent a tumorigenesis pathway that is shared with mucinous tumours of the appendix and ovary [6].

Urachal MCTLMP is a rare neoplasm arising from the epithelium of the urachus, with fewer than 50 cases described as such in the literature to date [3, 5–19]. The urachus is a fibrous midline structure that extends from the umbilicus to the anterosuperior aspect of the dome of the bladder, which is formed from remnants of the embryological allantois at 12 weeks of gestation, and involutes at birth to form the median umbilical ligament [20]. Incomplete atresia of the urachus, thought to occur in up to one‐third of adults, can give rise to a range of pathological conditions including urachal cyst, umbilical urachal sinus, vesicourachal diverticulum, patent urachus and urachal neoplasia [21]. The mean age at time of diagnosis of urachal MCTLMP is 50 years [3]. There is no predilection for male or female sex, and no clear risk factors for the condition have yet been identified [3]. The clinical presentation for urachal MCTLMP is highly variable and can feature urinary urgency, urinary frequency, dysuria, haematuria, mucusuria, abdominal pain, abdominal mass or umbilical discharge [16]. In many instances, however, the condition is discovered incidentally [16]. Urachal MCTLMP can be complicated by locally aggressive spread and pseudomyxoma peritonei and for this reason is routinely treated surgically: Most tumours are managed with partial cystectomy, with or without urachectomy and umbilectomy [18]. There are no reported cases of metastatic spread of urachal MCTLMP or mortality directly attributed to urachal MCTLMP [3].

## 2. Case Report

### 2.1. Patient History

A 59‐year‐old female was referred to the urology department following the incidental discovery of a bladder lesion identified on computed tomography studies, performed as part of surveillance for thyroid carcinoma diagnosed 6 months previously. Medical history was significant for Stage II follicular variant of papillary thyroid carcinoma, Type 2 diabetes mellitus, hypertension, dyslipidaemia and gastroesophageal reflux disease. Surgical history was significant for total thyroidectomy and total hysterectomy with bilateral ophorectomy. The patient was a current smoker with a 35‐pack‐year history but no family history of bladder cancer. On review at the urology department, the patient disclosed several months of urinary frequency and urinary urgency, without dysuria, haematuria, mucusuria or systemic symptoms. Physical examination was unremarkable, with nil appreciable tenderness to palpation, palpable mass or umbilical discharge or erythema. Full blood and serum chemistry studies were unremarkable.

**Figure 1:**
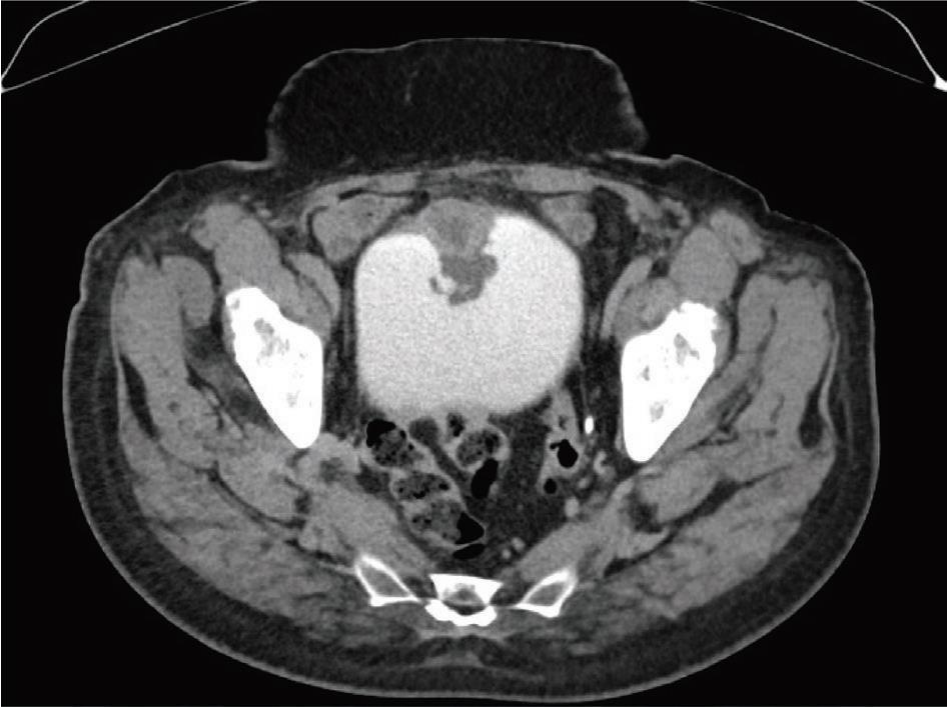
Computed tomography intravenous pyelography study (axial view).

**Figure 2:**
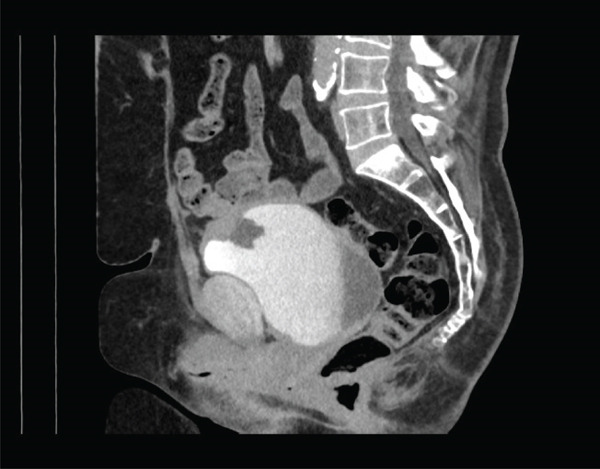
Computed tomography intravenous pyelography study (sagittal view).

Computed tomography intravenous pyelography was performed and demonstrated a localised area of bladder wall thickening at the anterosuperior aspect of the urinary bladder at the midline, with a central area of cystic change or necrosis. The area of bladder wall thickening measured 37.8 × 45.1 × 25.7 mm, and the central area of low‐density change measured 19.2 × 20.9 × 13.4 mm. The lesion was associated with several villous projections into the urinary bladder lumen. No para‐aortic, pelvic or inguinal lymphadenopathy was noted, and a differential diagnosis including urothelial lesion or complicated urachal remnant was suggested (Figures 1 and 2).

Rigid cystoscopy and transurethral biopsy of bladder tumour were subsequently performed. Cystoscopy directly visualised a lesion near the dome of the bladder with circumferential villous change and extrusion of mucus or slough. This lesion was biopsied, and histological review reported the specimen as most likely representing an area of intestinal metaplasia. No dysplasia or invasive malignancy was identified. Computed tomography chest, abdomen and pelvis with contrast, oesophagogastroduodenoscopy and colonoscopy performed at this time demonstrated no evidence of mass external to the bladder or urachus.

Flexible cystoscopy and open partial cystectomy were subsequently performed. Cystoscopy once again visualised a lesion near the dome of the bladder, and intraoperative cystoscopic transillumination of the circumference of the lesion facilitated elliptical resection of the urachal sheath, the distal urachal ligament, the lesion and the involved dome of the bladder. The resected tissue was submitted en bloc for histological evaluation. Repeat cystoscopy performed 9 months following open partial cystectomy demonstrated no evidence of lesion recurrence.

### 2.2. Findings

**Figure 3:**
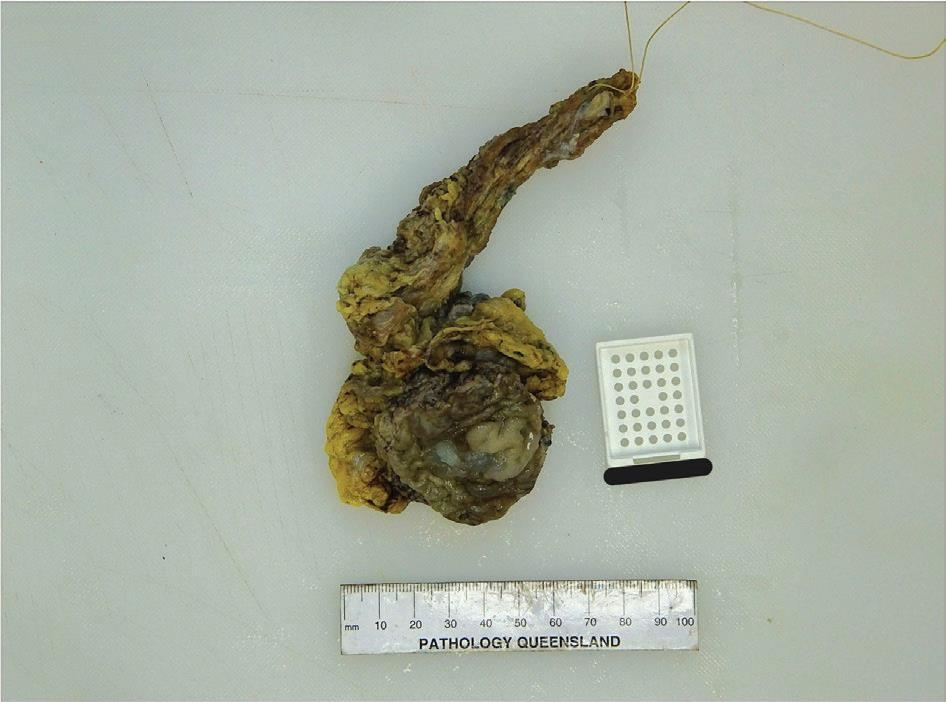
Gross specimen (suture designates umbilical edge).

**Figure 4:**
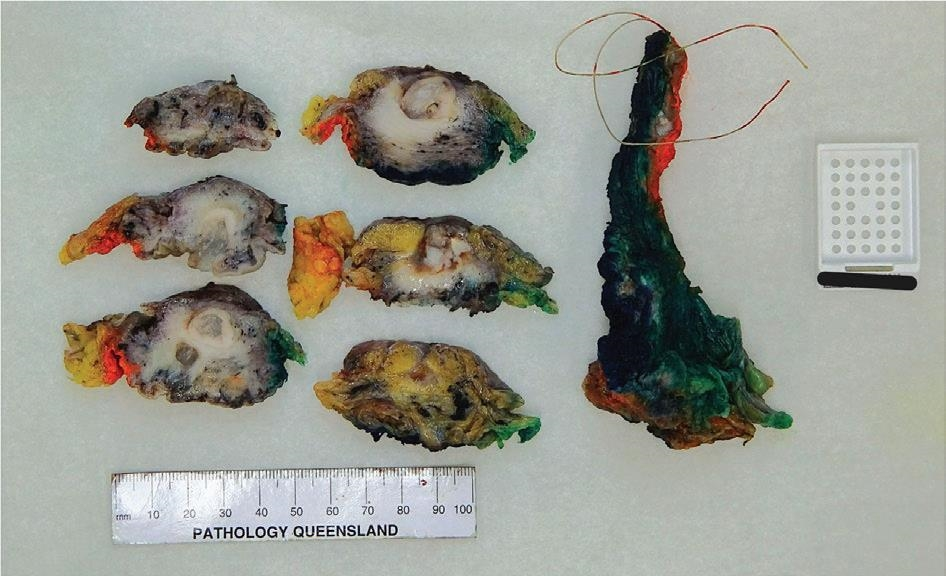
Gross specimen, inked and sectioned (suture designates umbilical edge, blue ink designates anterior aspect, black ink designates posterior aspect, orange ink designates right lateral aspect and green ink designates left lateral aspect).

The received specimen consisted of a partial cystectomy with en bloc distal urachal ligament and sheath measuring 155 × 65 × 40 mm. The portion representing the bladder dome measured 55 × 50 × 17 mm, and the portion representing ligamentous and soft tissue measured 110 × 20 × 10 mm. On the mucosal surface of the bladder, there was mucoid material. On sectioning, there was a dilated cystic lesion containing mucin surrounded by a wall of opaque tissue, 3 mm in thickness, which communicated with the mucosa posteriorly and tracked progressively deeper towards the serosa anteriorly. Tumour involvement of the serosa was not noted. Tumour involvement of the ligamentous and soft tissue was not noted. Macroscopically, the tumour appeared clear of all surgical margins (Figures 3 and 4).

**Figure 5:**
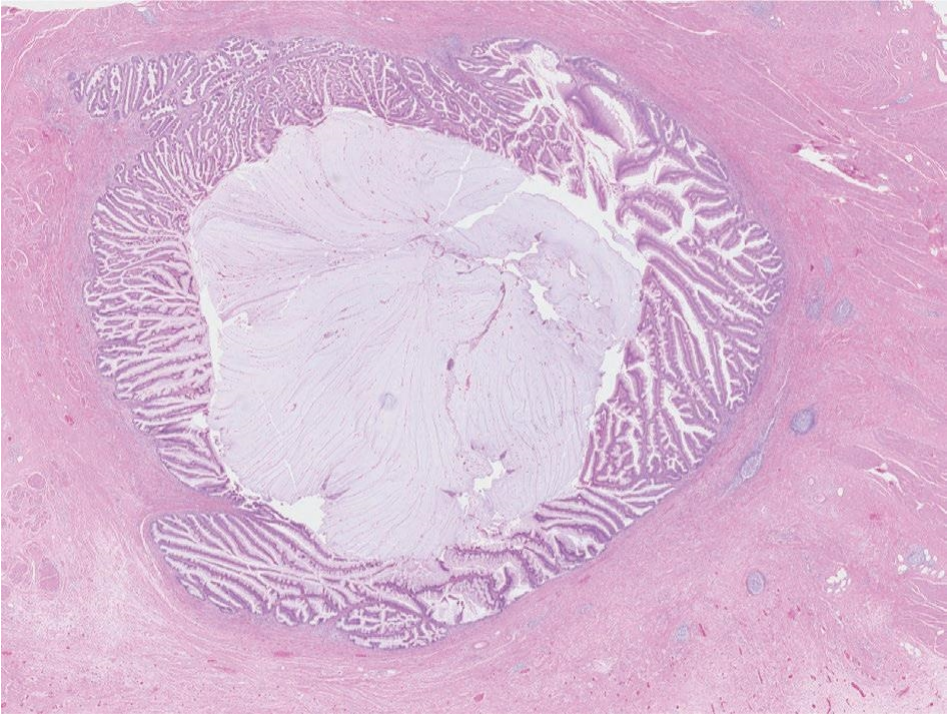
Full lesion centred in the muscularis propria of the urinary bladder; haematoxylin and eosin stain.

**Figure 6:**
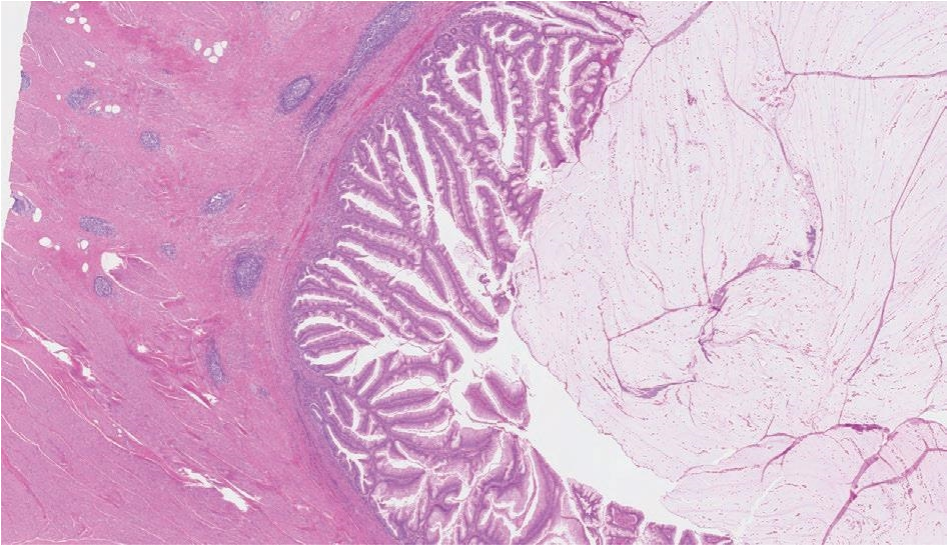
Urachal MCTLMP with mucin elaboration; no invasive malignancy; haematoxylin and eosin stain.

**Figure 7:**
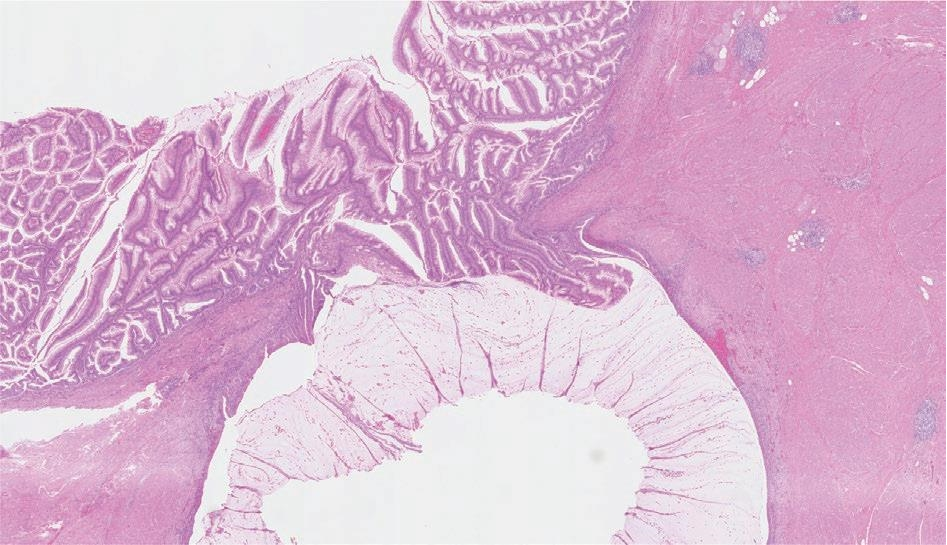
Urachal MCTLMP with mucin elaboration; no invasive malignancy; haematoxylin and eosin stain.

**Figure 8:**
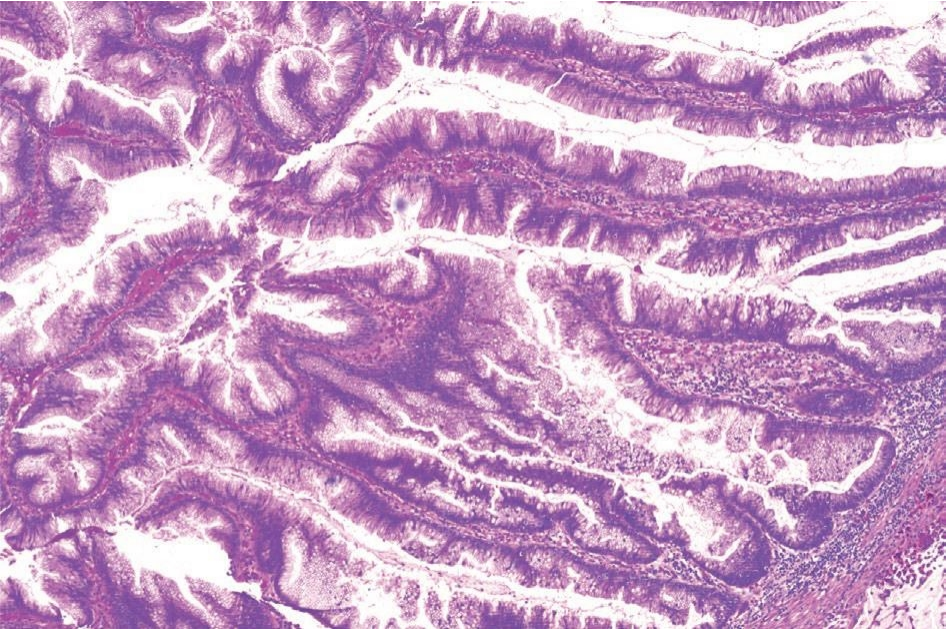
Urachal MCTLMP, villous architecture with low‐grade penicillate nuclei, mild pseudostratification and mild to moderate atypia; no invasive malignancy; haematoxylin and eosin stain.

**Figure 9:**
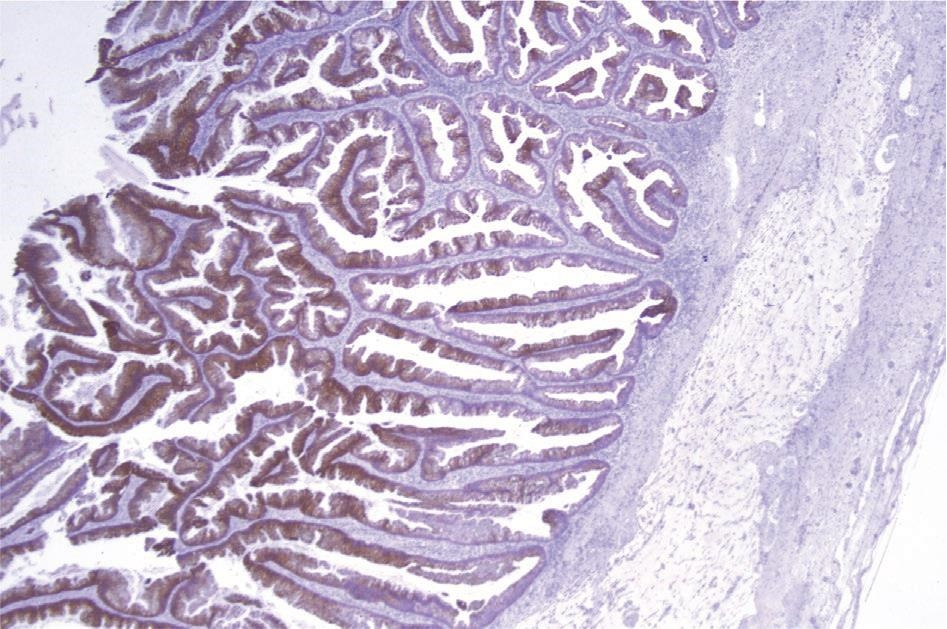
Urachal MCTLMP; CK20 immunohistochemical stain suggestive of intestinal immunophenotype.

**Figure 10:**
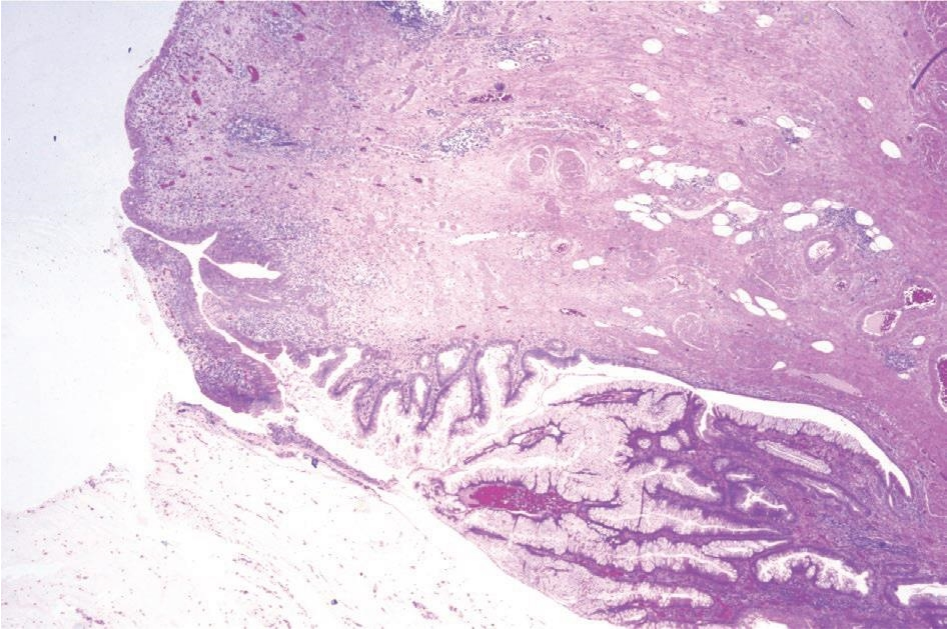
Urachal remnant with MCTLMP communicating with the urinary bladder surface; haematoxylin and eosin stain.

On microscopy, sections of the partial cystectomy specimen showed bladder wall in which there was a cystic villoglandular lesion centred in the muscularis propria in continuation with a cystic urachal remnant lined by urothelium. There was low‐grade dysplasia with the lesion showing villoglandular architecture with fibrovascular cores lined by pseudostratified columnar epithelium, crowding and nuclear hyperchromasia. There were no unequivocal malignant cytoarchitectural features characterised by high‐grade cytology, complex cribriform architecture, brisk mitosis or marked epithelial stratification. There was no stromal invasion. The lesion abutted the muscularis propria in a pushing manner with abundant elaborated mucin, fibrosis and inflammation. Mild nonspecific chronic inflammation was present in the superficial lamina propria. Sections of the distal urachal ligament and sheath specimen showed fibroconnective tissue and muscle with no patent urachal remnants. The histological features were noted to be reminiscent of a low‐grade appendiceal neoplasm or a borderline ovarian mucinous neoplasm. Changes were favoured to represent a urachal MCTLMP without intraepithelial carcinoma (Figures 5, 6, 7, 8, 9 and 10). The specimen was embedded entirely for histological examination, and MCTLMP was clear of all margins by greater than 5 mm.

Immunohistochemistry studies were performed, demonstrating positive staining with CDX2 and CK20, suggestive of an intestinal immunophenotype, and negative staining with PAX8, CK7 and ER, suggestive of an origin external to the female genital tract (Figure 9). Overall, immunohistochemical analysis was not of significant diagnostic value in this case, with the diagnosis clinched through histomorphological features and imaging studies.

## 3. Discussion

Urachal MCTLMP is a rare but clinically significant entity in the spectrum of urachal mucinous neoplasms spanning benign cystadenoma to invasive adenocarcinoma. Generalisations regarding the clinical course and prognosis of urachal MCTLMP are difficult to formulate, given the rarity of the condition. However, it is important to note that a proportion of reports of urachal cystadenoma and urachal cystadenocarcinoma prior to 2016 may in fact represent cases of urachal MCTLMP, owing to significant changes made in 2016 regarding the classification of urachal epithelial neoplasms in the World Health Organization Classification of Tumours [22]. These cases will not be considered here, although some of these cases have been previously examined by authors including Wang et al. [3]

A summary of 37 reports of urachal MCTLMP in the English‐language scientific literature to date is provided in Table 1, including the present case. The median age at time of diagnosis is 43 years, with a range of 26–80 years and an interquartile range of 38.5–59 years (*n* = 37). There is no significant predilection for female or male sex, with 19 cases described in female patients and 18 in male patients (*n* = 37). The median size of the tumour at time of diagnosis is 4 cm, with a range of 0.8–10 cm and an interquartile range of 2.1–7.3 cm (*n* = 32). All cases in which management was described were managed with resection of tumour (*n* = 32), with 27 cases involving partial cystectomy (84.4%), five cases involving umbilectomy (15.6%) and one case involving urachectomy (3.1%). One case was managed with transurethral resection of bladder tumour (3.1%). Of the cases in which the presenting complaint is noted (*n* = 35), 17 cases were discovered incidentally (48.6%), seven cases reported abdominal pain (20.0%), seven cases reported a palpable mass (20.0%), three cases reported haematuria (8.6%), three cases reported mucusuria (8.6%), two cases reported urinary urgency (5.7%) and isolated cases reported urinary frequency, dysuria or umbilical discharge (2.9%).

**Table 1:** Summary of reports of urachal MCTLMP in the English‐language scientific literature to date (F = female, M = male, NA = not available).

Reference	Age	Sex	Size (cm)	Treatment	Symptoms
Alatasi et al. [7]	43	M	7.6	Excision of tumour	Abdominal pain, mass
Amin et al. [6]	48	F	8	Mass excision/partial cystectomy and umbilectomy	Haematuria, mass
Amin et al. [6]	26	F	2	Mass excision/partial cystectomy	Mass
Amin et al. [6]	74	M	6.5	Excision of tumour and sigmoid colectomy	Incidental finding
Amin et al. [6]	72	M	0.8	Mass excision/partial cystectomy	Mucusuria
Amin et al. [6]	74	M	3	Mass excision/partial cystectomy	Haematuria
Amin et al. [6]	50	F	2.1	Mass excision/partial cystectomy	Mass
Amin et al. [6]	45	M	3.5	Mass excision/partial cystectomy	Abdominal pain, haematuria
Amin et al. [6]	58	F	1	Mass excision/partial cystectomy	Incidental finding
Amin et al. [6]	43	F	2.5	Mass excision/partial cystectomy	Incidental finding
Amin et al. [6]	40	F	6	Mass excision/partial cystectomy and umbilectomy	Incidental finding
Amin et al. [6]	80	F	2.5	Mass excision/partial cystectomy	Mucusuria
Amin et al. [6]	37	F	NA	NA	Incidental finding
Amin et al. [6]	29	F	NA	NA	Mass
Amin et al. [6]	42	F	8	Mass excision/partial cystectomy	Mass
Amin et al. [6]	42	F	6	NA	Mass
Amin et al. [6]	36	F	NA	NA	Incidental finding
Amin et al. [6]	39	M	6.5	Mass excision/partial cystectomy and umbilectomy	Umbilical discharge
Amin et al. [6]	57	M	2.8	Mass excision/partial cystectomy	NA
Amin et al. [6]	77	F	5.5	Mass excision/partial cystectomy	NA
Amin et al. [6]	43	M	7	Mass excision/partial cystectomy and umbilectomy	Incidental finding
Amin et al. [6]	26	M	8	Mass excision/partial cystectomy	Urgency, abdominal pain
Bashar et al. [8]	38	M	2.0	Mass excision/partial cystectomy	Incidental finding
Bogaerts et al. [9]	59	F	1.6	Mass excision/partial cystectomy	Incidental finding
Brennan et al. [10]	67	M	9	Mass excision/partial cystectomy	Incidental finding
Chahal et al. [11]	37	M	4	Partial cystectomy and left hydrocelectomy	Incidental finding
Chen et al. [12]	74	M	10	Mass excision/partial cystectomy	Abdominal pain
De Corte and Ramadhan [5]	54	M	3.5	Mass excision/partial cystectomy	Dysuria, abdominal pain
Kovacs and Pillai [13]	35	M	NA	NA	Incidental finding
Montemayor et al. [14]	43	F	NA	Mass excision/partial cystectomy, urachectomy, umbilectomy, bilateral salpingectomy and anterior peritonectomy	Abdominal pain
Moyo et al. [15]	40	F	9	Excision of tumour	Abdominal pain
Palial et al. [16]	59	M	1.7	Mass excision/partial cystectomy	Mucusuria, frequency, urgency
Pasternak et al. [17]	28	F	8	Excision of tumour	Incidental finding
Saint et al. [18]	70	M	2	Transurethral resection of bladder tumour	Incidental finding
Schmeusser et al. [19]	43	F	3.5	Mass excision/partial cystectomy	Incidental finding
Wang and Sule [3]	54	M	4	Mass excision/partial cystectomy	Incidental finding
Present case	59	F	2.1	Mass excision/partial cystectomy	Incidental finding

Urachal MCTLMP poses several challenges for the diagnosing pathologist. Urachal remnants are lined by urothelium which can undergo glandular metaplasia and subsequently give rise to glandular neoplasms, as seen in this case: Histologically, careful examination of the entire lesion is required to exclude invasive carcinoma. Other histological differential diagnoses include intestinal metaplasia, urothelial carcinoma with glandular differentiation, primary adenocarcinoma of the bladder and glandular tumours with secondary involvement of the urachus or bladder. Important keys to the diagnosis of urachal MCTLMP include the location of the lesion at the dome of the bladder at the midline (Figures 1 and 2), the location of the epicentre of the lesion in the muscularis propria of the bladder wall (Figure 5), the absence of widespread cystitis cystica beyond the dome of the bladder and the absence of a known primary glandular tumour arising outside of the bladder or urachus [4]. The presence of an associated urachal remnant is also supportive of, but not necessary for, the diagnosis of urachal MCTLMP (Figure 10) [4]. Urachal remnants can frequently communicate with the bladder surface, which was also seen in this case [4].

From a clinical perspective, this case report highlights the clinical importance of both the complete surgical resection of the tumour and the exclusion of secondary involvement of the bladder or urachus by a glandular tumour for the diagnosis of urachal MCTLMP. Open partial cystectomy was performed to exclude intraepithelial or invasive carcinoma, as well as provide definitive management for urachal MCTLMP, and computed tomography and endoscopic studies were performed to exclude secondary involvement of the urachus or bladder by a glandular tumour, such as adenocarcinoma of the colon or diffuse‐type gastric adenocarcinoma. Otherwise, this case report can be considered to represent an emerging ‘typical’ clinical trajectory of a patient with urachal MCTLMP. Namely, the condition was discovered incidentally, associated with mild symptomatology and managed with surgical resection of the tumour with partial cystectomy; the condition was diagnosed based on characteristic histomorphological features in conjunction with imaging studies, with limited utility of immunohistochemistry studies; and the case was not complicated by aggressive local spread or pseudomyxoma peritonei, with no local recurrence or metastasis following surgical resection. It should be noted, however, that patient follow‐up at the time of writing represents only a 10‐month period, and this significantly limits any inferences that can be made regarding longer term clinical trajectory. A multidisciplinary team approach and a full histopathological evaluation are crucial for proper diagnosis and management, with ongoing research to refine treatment guidelines and improve prognostication.

## 4. Conclusion

Urachal MCTLMP represents a rare borderline lesion in the spectrum of urachal mucinous neoplasms, spanning benign cystadenoma to malignant cystadenocarcinoma. The clinical presentation of urachal MCTLMP is highly variable, and ongoing longitudinal study is required to understand the longer term behaviour and clinical course of the condition. This case report describes an instance of urachal MCTLMP discovered incidentally in a 59‐year‐old female following the performance of imaging studies for an unrelated condition, which was associated with mild symptomatology and managed with surgical resection of the tumour and partial cystectomy, with no complications and no recurrence after 10 months of follow‐up. In particular, this case report highlights the clinical importance of both the complete surgical resection of tumour and the exclusion of secondary involvement of the bladder or urachus by a glandular tumour for the diagnosis of urachal MCTLMP and represents the emerging ‘typical’ clinical trajectory of a patient with urachal MCTLMP.

## Funding

No funding was received for this manuscript.

## Consent

Written informed consent was obtained from the participant included in this study.

## Conflicts of Interest

The authors declare no conflicts of interest.

## Data Availability

Data sharing is not applicable to this article as no datasets were generated or analysed during the current study.

## References

[1] WHO Classification of Tumours Editorial Board , WHO Classification of Tumours: Urinary and Male Genital Tumours, 2022, 5th edition, World Health Organization.

[2] Paner G. P. , Lopez-Beltran A. , Sirohi D. , and Amin M. B. , Updates in the Pathologic Diagnosis and Classification of Epithelial Neoplasms of Urachal Origin, Advances in Anatomic Pathology. (2016) 23, no. 2, 71–83, 10.1097/PAP.0000000000000110, 2-s2.0-84957937035, 26849813. 26849813

[3] Wang D. and Sule N. , Mucinous Cystadenoma of the Urachus and Review of Current Classification of Urachal Mucinous Cystic Neoplasms, Archives of Pathology & Laboratory Medicine. (2019) 143, no. 2, 258–263, 10.5858/arpa.2017-0319-RS, 2-s2.0-85060383003, 30398914. 30398914

[4] Taylor A. S. , Mehra R. , and Udager A. M. , Glandular Tumors of the Urachus and Urinary Bladder: A Practical Overview of a Broad Differential Diagnosis, Archives of Pathology & Laboratory Medicine. (2018) 142, no. 10, 1164–1176, 10.5858/arpa.2018-0206-RA, 2-s2.0-85054462721, 30281367. 30281367

[5] De Corte K. and Ramadhan A. , Exploring the Enigma of a Urachal Mucinous Cystic Tumor of Low Malignant Potential (MCTLMP): A Case Report and Literature Review, Cureus. (2025) 17, no. 2, e78562, 10.7759/cureus.78562, 40062171. 40062171 PMC11888570

[6] Amin M. B. , Smith S. C. , Eble J. N. , Choi W. L. W. , Tamboli P. , and Young R. , Glandular Neoplasms of the Urachus, American Journal of Surgical Pathology. (2014) 38, no. 8, 1033–1045, 10.1097/PAS.0000000000000250, 2-s2.0-84904460699. 25025366

[7] Alatasi S. , Murshed K. , Khalil I. A. , and Al Bozom I. , Urachal Mucinous Cystic Tumor of Low Malignant Potential in a 43-Year-Old Man: Case Report and Literature Review, 2025, Authorea Preprints, 10.22541/au.173735727.75873503/v1.

[8] Bashar A. , Aabdeen M. , George J. , McCrone R. , and El-Hassan M. , Urachal Mucinous Cystic Tumour of Low Malignant Potential: A Case Report With Literature Review, Cureus. (2025) 17, no. 8, e90905, 10.7759/cureus.90905, 40995298. 40995298 PMC12456959

[9] Bogaerts Q. , Vanthoor J. , Goethuys H. , and Raskin Y. , Cystoscopic and Robotic-Assisted Laparoscopic Excision of a Rare Urachus Neoplasm by Partial Cystectomy, Urology Video Journal. (2023) 17, 100209, 10.1016/j.urolvj.2023.100209.

[10] Brennan K. , Johnson P. , Curtis H. , and Arnason T. , Urachal Mucinous Cystic Tumor of Low Malignant Potential With Concurrent Sigmoid Colon Adenocarcinoma, Case Reports in Gastrointestinal Medicine. (2019) 2019, 1434838, 10.1155/2019/1434838, 31341685. 31341685 PMC6614953

[11] Chahal D. , Martens M. , and Kinahan J. , Mucinous Cystic Tumour of Low Malignant Potential Presenting in a Patient With Prior non-seminatous Germ Cell Tumour, Canadian Urological Association Journal. (2015) 9, no. 9-10, e750–e753, 10.5489/cuaj.2946, 2-s2.0-84944264479, 26664515. 26664515 PMC4662440

[12] Chen L. , Di M. , Sun L. , and Fu Q. , Rare Urachal Mucinous Cystic Tumor of Low Malignant Potential With Peritoneal Pseudomyxoma: A Case Report, Experimental and Therapeutic Medicine. (2023) 26, no. 6, 10.3892/etm.2023.12254, 37941591. PMC10628641 37941591

[13] Kovacs T. and Pillai S. , Urachal Mucinous Cystic Neoplasm of Low Malignant Potential: A Rare Entity, Pathology. (2023) 55, no. 1, s61–s62, 10.1016/j.pathol.2022.12.201.

[14] Montemayor J.-A. S. , Hilvano-Cabungcal A. M. , Lapitan M. C. M. , Sotalbo K. C. J. , and Manibo M. V. M. , Ruptured Urachal Mucinous Cystic Tumour of Lower Malignant Potential: A Case Report and Review of Literature, Philippine Journal of Urology. (2024) 34, no. 1, 26–31, http://pjuonline.com/index.php/pju/article/view/181.

[15] Moyo M. T. G. , Dirilenoğlu F. , and Köy Y. , Urachal Mucinous Cystic Tumor of Low Malignant Potential: A Report of a Rare Case With Literature Review, Surgical and Experimental Pathology. (2023) 6, no. 1, 10.1186/s42047-023-00143-z.

[16] Palial K. , Yang B. , Charlesworth P. J. S. , Lewis C. E. , Browning L. , and Verrill C. , A Rare Case of a Urachal Mucinous Cystic Tumour of Low Malignant Potential, Cancer Studies and Molecular Medicine–Open Journal. (2018) 4, no. 1, 5–9, 10.17140/CSMMOJ-4-122.

[17] Pasternak M. C. , Black J. D. , Buza N. , Azodi M. , and Gariepy A. , An Unexpected Mass of the Urachus: A Case Report, American Journal of Obstetrics and Gynecology. (2014) 211, no. 4, e1–e3, 10.1016/j.ajog.2014.06.011, 2-s2.0-84907687091, 24912094. 24912094

[18] Saint F. , Renard C. , Cordonnier C. , Tesson J. R. , Priam A. , and Amant C. , Endoscopic Management of Urachal Mucinous Cystic Tumor of Low Malignant Potential, World Journal of Surgery and Research. (2024) 7, https://www.surgeryresearchjournal.com/open-access/endoscopic-management-of-urachal-mucinous-cystic-tumor-of-low-malignant-9728.pdf.

[19] Schmeusser B. , Wiedemer J. , Obery D. , Buckley K. , and Yu M. , Urachal Mucinous Cystic Tumor of Low Malignant Potential in a Polymorbid Female: A Case Report and Review of the Literature, International Cancer Conference Journal. (2022) 11, no. 2, 104–108, 10.1007/s13691-021-00530-x. 35402128 PMC8938540

[20] Moore K. L. , The Developing Human: Clinically Oriented Embryology, 2019, 11th edition, Saunders.

[21] Briggs K. B. and Rentea R. M. , Patent Urachus, Stat Pearls, 2023, StatPearls Publishing. 32491655

[22] WHO Classification of Tumours Editorial Board , WHO Classification of Tumours: Urinary and Male Genital Tumours, 2016, 4th edition, World Health Organization.

